# Developing an algorithm to identify individuals with psychosis in secondary care in England: application using the Mental Health Services Data Set

**DOI:** 10.1192/bjo.2024.853

**Published:** 2025-02-27

**Authors:** Claire de Oliveira, Maria Ana Matias, María José Aragon Aragon, Misael Anaya Montes, David Osborn, Rowena Jacobs

**Affiliations:** Centre for Health Economics, University of York, York, UK; Hull York Medical School, Hull and York, UK; Institute for Mental Health Policy Research, Centre for Addiction and Mental Health, Toronto, Ontario, Canada; Institute of Health Policy, Management and Evaluation, University of Toronto, Toronto, Ontario, Canada; ICES, Toronto, Ontario, Canada; HCD Economics, Las Palmas, Spain; Division of Psychiatry, University College London, London, UK

**Keywords:** Administrative data, algorithms, psychosis, Mental Health Services Data Set, schizophrenia

## Abstract

**Background:**

There is currently no definitive method for identifying individuals with psychosis in secondary care on a population-level using administrative healthcare data from England.

**Aims:**

To develop various algorithms to identify individuals with psychosis in the Mental Health Services Data Set (MHSDS), guided by national estimates of the prevalence of psychosis.

**Method:**

Using a combination of data elements in the MHSDS for financial years 2017–2018 and 2018–2019 (mental health cluster (a way to describe and classify a group of individuals with similar characteristics), Health of the Nation Outcome Scale (HoNOS) scores, reason for referral, primary diagnosis, first-episode psychosis flag, early intervention in psychosis team flag), we developed 12 unique algorithms to detect individuals with psychosis seen in secondary care. The resulting numbers were then compared with national estimates of the prevalence of psychosis to ascertain whether they were reasonable or not.

**Results:**

The 12 algorithms produced 99 204–138 516 and 107 545–134 954 cases of psychosis for financial years 2017–2018 and 2018–2019, respectively, in line with national prevalence estimates. The numbers of cases of psychosis identified by the different algorithms differed according to the type and number (3–6) of data elements used. Most algorithms identified the same core of patients.

**Conclusions:**

The MHSDS can be used to identify individuals with psychosis in secondary care in England. Users can employ several algorithms to do so, depending on the objective of their analysis and their preference regarding the data elements employed. These algorithms could be used for surveillance, research and/or policy purposes.

Administrative healthcare data represent a valuable source of information that can be used for different purposes, including tracking patients’ interactions with the healthcare system. However, identifying individuals with a long-term condition such as psychosis from these data can be challenging, as most administrative data-sets were not created for surveillance, research, and/or policy purposes. For example, it is relatively easy to identify individuals diagnosed with psychosis in primary care data in England using Read codes (a clinical terminology system used to code multiple patient phenomena, such as diagnoses, ethnicity and religion, and social circumstances)^1^ and/or SNOMED codes (computer-processable medical terms providing codes, terms, synonyms and definitions used in clinical documentation and reporting) (https://www.snomed.org/), given the implementation of Quality and Outcomes Framework, a voluntary annual reward and incentive programme for all general practitioners in England).^2^ However, there is currently no definitive method to identify individuals with psychosis in secondary care data, given that diagnostic coding is not mandatory in the data submitted to the Mental Health Services Data Set (MHSDS), resulting in high levels of missing data.

Prior studies that have attempted to identify individuals with schizophrenia or psychosis have either relied solely on hospital data,^3^ which can accurately identify individuals with schizophrenia or psychosis but only captures severe cases, or insurance data, such as US Medicaid data,^4^ which only captures a subgroup of this population. Both approaches are problematic, as they do not produce population-based samples. More recently, other studies have used both hospital and out-patient administrative data to ascertain cases of psychosis at the population level, thereby producing more representative samples. Using administrative healthcare data from Ontario, Canada, one study developed and compared eight algorithms to identify individuals with chronic psychotic disorders using hospital admission records and physician billing claims linked to diagnostic information abstracted from clinical records, defined as the reference standard.^5^ The researchers found that using only hospital admission records yielded the highest specificity and the highest positive predictive value, whereas using physician billing claims in addition to hospital admission records increased sensitivity but decreased specificity and the positive predictive value. However, the proposed algorithms did not capture cases diagnosed by psychologists, for example. Using data from France, another study examined claims data for the reimbursement of ambulatory care in private practice (e.g. medical consultations and procedures and medication) linked to national hospital discharge databases.^6^ The authors developed three algorithms using different variations in diagnoses (principal or associated) and hospital admission records, out-patient care data and antipsychotic medication claims. Although the algorithms produced population-based estimates of prevalence rates of schizophrenia and psychosis in line with reported estimates from systematic reviews, the study did not validate the algorithms or indicate which of the three was the preferred one. Finally, another study used data on diagnoses recorded in in-patient and community mental health records from Australia and compared these with linked data from the Australian National Survey of Psychosis on psychotic disorders obtained from semi-structured clinical interviews of individuals.^7^ The authors compared the relative performance of four algorithms. Overall, agreement between the administrative and survey data was modest; however, the results may have been biased, as participants were not a random sample of patients and underwent diagnostic interview only after screening positive for probable psychosis.

The objective of the present study was to develop various algorithms to identify individuals with psychosis, guided by national estimates, using the MHSDS, the main administrative data-set that captures all secondary mental healthcare activity in England. In England, healthcare is mainly provided by the National Health Service (NHS), which provides healthcare to all permanent residents, free of charge. The NHS provides primary and secondary care: primary care is provided by general practitioners, who can refer patients to further services, where required; whereas secondary care covers planned or elective care, urgent and emergency care, and mental healthcare. Secondary mental healthcare includes care provided in hospitals, some psychological well-being services, care provided by community mental health teams, and care provided by crisis resolution and home treatment teams. This is the first attempt to undertake this exercise using data from England. The resulting algorithms are expected to help identify individuals with psychosis at the national level and thus inform surveillance-, research- and/or policy-related activities.

## Method

### Data source

The MHSDS includes data on mental healthcare services provided in secondary care (i.e. hospitals, some psychological well-being services, community mental health teams, and crisis resolution and home treatment teams) to children, youth and adults in England.^8^ The data-set was designed to capture information on anyone thought to be suffering from a mental illness and in receipt of specialist mental healthcare in settings partially or wholly funded by the NHS, the publicly funded healthcare system in England. The MHSDS versions 4 and 5 include data on secondary care mental health services provided in hospitals, community settings and out-patient clinics. They provide information on the type of care received, length of care, information on contacts with mental health and social care professionals, and measures of health and social functioning.

The MHSDS comprises nine smaller sub-data-sets, which contain specific information regarding patients and the type of care provided: (a) patient details; (b) clinical coded terminology; (c) care clusters; (d) referrals; (e) care contact and activities; (f) group sessions; (g) hospital provider spells; (h) mental health act legal status classification period; and (i) care programme approach episodes. There are currently six data elements and/or variables in the MHSDS, which can be used to identify patients with psychosis: (a) mental health clusters and child and adolescent mental health needs-based groupings; (b) HoNOS scores; (c) reason for referral; (d) primary diagnosis; (e) first-episode psychosis flag; and (f) early intervention in psychosis (EIP) team flag. Supplementary Table 1 available at https://doi.org/10.1192/bjo.2024.853 provides a fuller explanation of each data element and/or variable. In this analysis, psychosis included all cases of schizophrenia, schizoaffective disorder and other psychotic disorders (e.g. psychosis not otherwise specified) but not other disorders that may involve psychotic symptoms, such as bipolar disorder, psychotic depression or psychotic dementia.

### Data elements and/or variables used to identify psychosis

Some data elements and/or variables can be used independently (i.e. on their own) to identify cases of psychosis, whereas others may need to be used jointly with other variables to increase case finding. Therefore, it is important to understand the limitations of each one *a priori*.

- A mental health cluster is a way of describing and classifying a group of individuals with similar characteristics identified through a holistic assessment by a healthcare professional and in some instances rated using the mental health clustering tool.^9^ The mental health clustering tool is a needs assessment tool designed to rate the care needs of patients and thus is not meant to be a diagnostic tool. Furthermore, for many clusters, the likely primary diagnosis can include cases of both psychosis and bipolar disorder, which adds some uncertainty when using the cluster variable alone to identify individuals with psychosis. The same rationale applies to the child and adolescent mental health needs-based groupings.
- The HoNOS item ‘problems associated with hallucinations and delusions’ (item 6, 7 or 8 depending on the HoNOS version) with a score ≥ 3 can be used to identify cases of psychosis; however, because this item may also select individuals with other conditions such as personality disorder and post-traumatic stress disorder, who can also present with psychotic symptoms, it is not sufficient to identify all cases of psychosis. Therefore, these items are not reliable for identifying cases of psychosis and should be used jointly with other variables to ascertain cases of psychosis.
- The reason for referral is defined as the primary presenting condition or symptom for which the patient is referred to a mental health service. Individuals with psychosis can be identified using either code 01 ((suspected) first-episode psychosis) or code 02 (ongoing or recurrent psychosis). Using code 01 alone could potentially capture unconfirmed cases of first-episode psychosis; thus, it should be used with other variables to confirm true cases of first-episode psychosis. Code 02 can probably be used independently to ascertain cases of psychosis. There is also code 18 (in crisis), which does not have any diagnosis attached to it and can be used in many situations, such as cases of psychotic crisis; therefore, this code must be used in conjunction with other variables to confirm whether it refers to a case of psychosis.
- The primary diagnosis is defined as the main condition treated or investigated during an episode of care and, where there is no definitive diagnosis, the main symptom, abnormal findings or problem. This variable can probably be used independently to ascertain cases of psychosis where there is a definitive diagnosis.
- The EIP data contain information on people with a first episode of psychosis who have accessed care or are waiting for treatment but have had a first contact with the EIP team. Based on this information, an administrative flag is created to indicate contact with the EIP team. This variable can probably be used independently to ascertain cases of first-episode psychosis only.
- Finally, the EIP teams are multidisciplinary teams set up to seek, identify and reduce treatment delays at the onset of psychosis and promote recovery by reducing the probability of relapse following a first episode of psychosis. Based on contact with the EIP team, an administrative flag is created. However, on its own, this variable cannot be used to identify cases of psychosis, because diagnostic classification based on psychopathology at first contact with EIP teams may take time to validate; thus, it should be used jointly with the ‘reason for referral’ variable code 18.

See Table 1 for further details on the data elements and/or variables used to identify psychosis in the MHSDS.

**Table 1:**
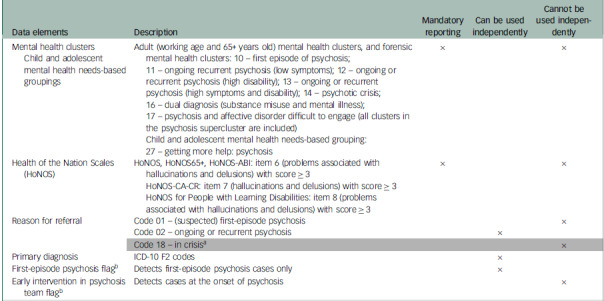
Data elements used (independently or jointly with other data elements) to identify patients with a diagnosis of psychosis in the Mental Health Services Data Set

ABI, acquired brain injury; CA-CR, child and adolescent clinician-rated.

a. This code cannot be used on its own to identify cases with psychosis; it must be used jointly with the early intervention in psychosis flag.

b. Flags are derived from the administrative data and indicate presence of a visit.

### Data assumptions

It is also important to impose age restrictions when selecting cases of psychosis. For incident cases, individuals between the ages of 14 and 65 years at diagnosis were selected.^10^ The onset of psychosis rarely occurs before the age of 14;^11^ therefore, all individuals under 14 years of age were excluded from the analysis. To avoid including cases of dementia or psychotic dementia among older individuals (i.e. those aged 55 to 65 years old), all cases in which this diagnosis occurred at these ages were excluded.^12^ For prevalent cases, all individuals between the ages of 14 and 105 years were considered. In addition, some simplifying assumptions were made. For example, in many instances, researchers try to match patients to providers as a method to link the different sub-data-sets within the MHSDS; as the main objective was to identify individuals with psychosis and not to examine their healthcare utilisation, patient-provider matches were not accounted for in this analysis. Potential contradictions within algorithms were also ignored; for example, if a patient was assigned to more than one mental health cluster, of which one indicated psychosis and another did not, the patient was classified as having a diagnosis of psychosis (the only exceptions were cases of first-episode psychosis between the ages of 55 and 65 years, where individuals had a concurrent diagnosis of dementia or psychotic depression).^a^

### External validation using national prevalence data for psychosis

Finally, we used national estimates of the prevalence of psychosis to help guide the analysis and ascertain whether the numbers from our proposed algorithms were reasonable or not. The prevalence data were derived from various sources to determine an interval of potential number of psychosis cases treated in secondary care. According to the 2014 Adult Psychiatric Morbidity Survey, the estimated prevalence of psychosis in England among adults aged 16 and over is 0.7%.^13^ Other work has found that 69% of people with severe mental illness were seen in secondary care between 2012 and 2014.^14^ It has also been found that 64% of those with severe mental illness seen in primary care between 2000 and 2018 had psychosis,^15^ and 43% of people with psychosis were seen in both primary and secondary care between 2012 and 2014.^14^ Combining these numbers and using population estimates from the Office for National Statistics,^16^ we obtained a range of 96 234–143 232 adults aged 16 and over with psychosis treated in secondary care in England in 2014, corresponding to an annual treated prevalence rate of psychosis between 0.22% and 0.33%.

### Algorithm development

Based on the available data elements and/or variables in the MHSDS, we developed algorithms and applied them to financial years 2017–2018 and 2018–2019 of the MHSDS. The algorithms were developed with two goals in mind: to obtain a number of psychosis cases in line with the proposed range of treated prevalence rates in both years; and to increase the sensitivity of cases selected (i.e. increase the number of true cases of psychosis). Given that no single variable would be likely to produce a large enough sample of true cases of psychosis with certainty in line with the proposed treated prevalence rates (Supplementary Table 2), we developed various algorithms based on several combinations of variables and/or data elements that can be used independently (e.g. primary diagnosis, reason for referral code 02, first-episode psychosis flag) using ‘OR’, as well as variables and/or data elements that must be used jointly with other variables (e.g. EIP team flag, mental health clusters, HoNOS, reason for referral codes 01 and 18) using ‘AND.’ Double counting was accounted for where applicable. Given all possible combinations for these variables, we obtained 33 algorithms (available upon request). However, only 12 algorithms produced numbers within the expected range of treated prevalence rates (0.22–0.33%) for each year of the analysis (i.e. between 98 351–146 383 in 2017–2018 and 98 892–147 188 in 2018–2019 based on population estimates of persons of both sexes between the ages of 16 and 90 years for local authorities in England obtained from the Office for National Statistics^16^). In addition, we examined the level of agreement among the 12 proposed algorithms to determine whether there was a core set of patients who were flagged by all algorithms.

## Results

In 2018–2019, the total number of patients in contact with secondary care in the MHSDS was 2 378 247; in 2017–2018, it was 2 220 812. Table 2 shows the 12 proposed algorithms and the number of patients obtained for each one (in 2018–2019 and 2017–2018, the total numbers of patients with at least one data element were 280 396 and 275 495, respectively). Table 3 shows the number of algorithms that identify the same core set of patients. The number of potential patients with a diagnosis of psychosis in 2018–2019 varied between 107 545 and 146 832. Most algorithms identified the same core of patients; for example, in 2018–2019, all 12 algorithms identified the same 48 681 patients.

**Table 2:** Proposed algorithms to identify patients with a diagnosis of psychosis in the Mental Health Services Data Set and respective numbers of patients for financial years 2017–2018 and 2018–2019

Algorithm	Definition^a^	2017–2018	2018–2019
1	Primary diagnosis OR reason for referral = code 02 OR FEP flag OR EIP team flag	119 078	134 964
2	Primary diagnosis OR reason for referral = code 02 OR FEP flag OR (EIP team flag AND mental health clusters)	107 031	122 426
3	Primary diagnosis OR reason for referral = code 02 OR FEP flag OR (EIP team flag AND HoNOS)	100 508	114 742
4	Primary diagnosis OR reason for referral = code 02 OR FEP flag OR (EIP team flag AND mental health clusters AND HoNOS)	99 888	114 081
5	Primary diagnosis OR reason for referral = code 02 OR FEP flag OR (mental health clusters AND HoNOS)	134 140	146 997
6	Primary diagnosis OR FEP flag OR EIP team flag	99 204	107 545
7	Primary diagnosis OR FEP flag OR EIP team flag OR (mental health clusters AND HoNOS)	138 516	146 832
8	Primary diagnosis OR FEP flag OR EIP team flag OR (mental health clusters AND (reason for referral = code 01 OR = code 02))	110 603	121 394
9	Primary diagnosis OR FEP flag OR EIP team flag OR (HoNOS AND (reason for referral = code 01 OR = code 02))	105 924	115 699
10	Primary diagnosis OR FEP flag OR EIP team flag OR (mental health clusters AND HoNOS AND (reason for referral = code 01 OR = code 02))	104 843	114 388
11	Primary diagnosis OR FEP flag OR (mental health clusters AND HoNOS)	117 598	123 264
12	Reason for referral = code 02 OR FEP flag OR EIP team flag OR (mental health clusters AND HoNOS)	116 538	132 584

FEP, first-episode psychosis; EIP, early intervention in psychosis; HoNOS, Health of Nation Outcome Scales.

a. Mental health clusters include all clusters (adult (working age and 65+ years old) and forensic) and child and adolescent mental health needs. HoNOS includes HoNOS for working-age adults, HoNOS 65+ (Older Adults), HoNOS for Acquired Brain Injury, HoNOS for Children and Adolescents – Clinician Rated, and HoNOS for People with Learning Disabilities.

**Table 3:** Numbers of algorithms identifying the same patients using the Mental Health Services Data Set for financial years 2017–2018 and 2018–2019

	Number of patients	
Number of algorithms	2017–2018	2018–2019
1	951	654
4	33 673	32 444
6	8924	13 385
7	17 896	20 012
8	7763	9006
11	43 580	44 352
12	41 494	48 681

Algorithms 5 (primary diagnosis OR reason for referral = code 02 OR first-episode psychosis flag OR (mental health clusters AND HoNOS); *n* = 146 997) and 7 (primary diagnosis OR first-episode psychosis flag OR EIP team flag OR (mental health clusters AND HoNOS); *n* = 146 832) produced the largest number of individuals. Algorithm 6 (primary diagnosis OR first-episode psychosis flag OR EIP team flag; *n* = 107 545) made use of the smallest number of variables that could be used independently (in this case, three variables). Other algorithms that required few variables (e.g., four variables) included algorithms 1 (*n* = 134 964) and 11 (*n* = 123 264). Algorithm 1 was the only algorithm that maximised the number of cases using the fewest variables (i.e. four variables); it also used all four variables that could be used independently to identify cases of psychosis.

Data for financial year 2017–2018 provided similar numbers – algorithms 5 and 7 produced 134 140 and 138 516 patients, whereas algorithms 6, 1 and 11 produced 99 204, 119 078 and 117 598, respectively. Thus, the findings for financial year 2017–2018 were similar to those for financial year 2018–2019.

## Discussion

Given the structure of the MHSDS, it can be challenging to accurately identify individuals with specific mental health conditions from the data. As an exemplar, this analysis developed a series of algorithms to identify individuals with psychosis who were treated in secondary care in England. Using different combinations of existing data elements and guided by a plausible range of national estimates of psychosis cases treated in secondary care, we derived 12 algorithms, all but one of which made use of the primary diagnosis variable. Among these, we found that most algorithms captured the same individuals, increasing our confidence in terms of correctly identifying cases of psychosis. Depending on users’ preferences (e.g. to obtain the largest sample possible, to use the smallest number of variables possible) and purposes (surveillance, research, policy), there are several options. If users want to obtain the largest sample possible (within the suggested prevalence range) and use the smallest number of variables possible, algorithm 1 would be the preferred one. This algorithm produces a sample of 119 078 and 134 964 individuals in financial years 2017–2018 and 2018–2019, respectively, and makes uses of the ‘primary diagnosis,’ ‘reason for referral (code 02 – ongoing or recurrent psychosis),’ ‘first-episode psychosis flag’ and ‘early intervention psychosis team flag’ variables, all of which, in principle, can be used independently to select cases of psychosis. However, the choice will depend on which variables users believe to be the best when identifying individuals with psychosis seen in secondary care and the objective of their analysis (e.g. examining only incident cases). For example, some users may believe that the ‘reason for referral’ variable could be subject to measurement error, as it will probably be based on the best available information found in the referral.

To our knowledge, this is the first study to develop algorithms to detect cases of psychosis using secondary data from England, while addressing the limitations of previous algorithms developed elsewhere for the same purpose. For example, one study developed algorithms using hospital admission records and physician billing claims from Ontario, Canada, but did not capture cases diagnosed by psychologists.^5^ Another study used hospital admission records, out-patient care data and antipsychotic medication claims from France but did not validate the algorithms or indicate which was the preferred one.^6^ Finally, another study developed algorithms using health records and survey data from Australia but used a probably biased sample of patients. The algorithms proposed in this work represent an improvement over previous work: they use both in-patient and out-patient data, capturing cases diagnosed and treated by physicians, psychiatrists and psychologists, as well as both severe and less severe cases of psychosis, enabling them to produce population-level estimates; they have also been validated using national estimates on the prevalence of psychosis.

However, this study was not without limitations. There may have been individuals who were classified by the algorithms as having psychosis when in fact they did not have the disorder and vice versa. The algorithms are only applicable to the MHSDS version 4 (and probably future versions), not previous versions of the MHSDS, such as the Mental Health Minimum Data Set and the Mental Health and Learning Disabilities Data Set, as the structure of these data-sets differs from that of the MHSDS. Furthermore, it is important to note that the use of mental health clusters will be phased out in the future, as the current cluster model has been found to not be fit for purpose.^17^ Moving forward, the fields that will be mandatory for trusts to submit to the MHSDS will be diagnosis, reason for referral and team type for adults and needs for children and youth. Thus, the algorithms may need to be amended in the future. Nonetheless, given that none of the recommended algorithms makes use of the mental health clusters, this change is likely to have little impact on the recommended algorithms. In addition, given the multitude of algorithms proposed in this analysis, it was not possible to provide a gold standard (although some suggestions were provided in terms of which algorithms to use under which circumstances). Finally, owing to barriers to accessing chart records and primary care data linked to secondary care data, it was not possible to validate these algorithms against an external data source. This should be explored in future research. Nonetheless, national estimates of psychosis were used to help validate the prevalence numbers obtained.

It has been announced that the MHSDS will be used for commissioning purposes in England;^18^ as a result, the importance of this database is likely to grow over time, and both the quality of the data reporting and the accuracy of the algorithms are expected to improve. Surveillance data are important to measure and monitor healthcare activity among patients; resulting outputs can be used for prevention, planning and resource allocation, and policy purposes. The use of these algorithms will enable population-level surveillance of outcomes among this patient population, as well as examination of their healthcare utilisation, at minimal cost. For example, Public Health England, now the Office for Health Improvements and Disparities, developed an indicator of excess mortality for people with severe mental illness using the MHSDS^19^ but was unable to correctly identify relevant cases. These algorithms should be able to support this type of work. Moreover, the algorithms will help support much-needed high-quality research on this vulnerable patient population.

The choice of which algorithm to employ will depend on the purpose of the analysis. Nonetheless, based on our findings, the algorithm that makes use of all data elements that can be used independently to identify cases of psychosis (i.e. primary diagnosis, reason for referral code 02 – ongoing or recurrent psychosis, first-episode psychosis flag and early intervention psychosis team flag) and maximises the number of cases is likely to be the preferred one. Future research should seek to validate these algorithms using chart data and/or other independent data sources, as well as using the MHSDS to develop similar algorithms for other mental disorders. Improvements in the quality of diagnostic coding in future may partially negate the need for algorithm development; however, it is also important to note that early intervention services staff may be reluctant to provide a diagnosis to patients in the early stages of diagnostic assessment.

## Supporting information

de Oliveira et al. supplementary material de Oliveira et al. supplementary material

## Data Availability

This work uses data provided by patients and collected by the National Health Service (NHS) as part of their care and support. The MHSDS is copyright © 2017/18–2018/19, NHS England. Re-used with the permission of NHS England. All rights reserved.

## Footnotes

a In 2018–2019, 23 147 of 200 062 patients (11.6%) were assigned a mental health cluster indicating psychosis as well as another cluster that did not indicate psychosis. The figures for 2017–2018 were similar – 23 576 of 201 240 patients (11.7%).
