# Supplementary material for: Developing an algorithm to identify individuals with psychosis in secondary care in England: application using the Mental Health Services Data Set
Source: BJPsych Open. 2025 Feb 27;11(2):e37. doi: 10.1192/bjo.2024.853 (PMC12001952; doi:10.1192/bjo.2024.853)
Supplement: de Oliveira et al. supplementary material [file S2056472424008536sup001.docx]

**Appendix**

**Table A1.** Description of data elements/variables in the Mental Health Services Data Set used to identify psychosis

| **Data element/variable** | **Description** |
| --- | --- |
| Mental health clusters  Child and adolescent mental health needs-based groupings | A mental health cluster is a global description of a group of individuals with similar characteristics identified through a holistic assessment and then rated using the mental health clustering tool. In turn, the mental health clustering tool is a needs assessment tool designed to rate the care needs of a given patient, based on a series of 18 rating scales. It incorporates items from the Health of the Nation Outcome Scale and the Summary of Assessments of Risk and Need to provide the information necessary to allocate individuals to clusters. Clusters are a means of categorising adult service users into 1 of 20 categories with similar levels of need, which, in turn, are categorised into 1 of 3 super-clusters, one of which is psychosis. There are several versions of the mental health clusters: working age adult mental health clusters, adults 65+ mental health clusters, and forensic mental health clusters.  Child and adolescent mental health needs-based groupings are 19 groupings that categorise the need for advice or help of children, young people and/or families referred to a service. |
| Health of the Nation Outcome Scale (HoNOS) | The HoNOS is an internationally recognised outcome measure developed by the Royal College of Psychiatrists Research Unit to measure health and social functioning outcomes in mental health services. Its aim is to produce a brief measure capable of being completed routinely by clinicians and recorded as part of a minimum mental health dataset. The first 12 items of the mental health clustering tool are HoNOS items. There are several versions of the HoNOS: working age adults HoNOS, adults aged 65+ HoNOS, acquired brain injury HoNOS, children and adolescent clinician-rated scale HoNOS, and learning disabilities HoNOS. |
| Reason for referral | The primary reason for referral is defined as the primary presenting condition or symptom for which the patient was referred to a mental health service. |
| Primary diagnosis | The primary diagnosis is defined as the main condition treated or investigated during the episode of care and, where there is no definitive diagnosis, the main symptom, abnormal findings, or problem. |
| First episode psychosis flag | The Early Intervention in Psychosis data contains information on people with a first episode of psychosis who accessed or are waiting for treatment. |
| Early intervention in psychosis team flag | The Early Intervention in Psychosis teams are multi-disciplinary teams set up to seek, identify, and reduce treatment delays at the onset of psychosis and promote recovery by reducing the probability of relapse following a first episode of psychosis. Patients with psychosis are likely to be referred to this team. |

**Table A2.** Number of patients identified using each psychosis-related data element in the Mental Health Services Data Set for financial years 2017/18 and 2018/19

|  | **Number of patients** | |
| --- | --- | --- |
| **Data elements** | **2017/18** | **2018/19** |
| **Mental health clusters** (including working age adults, adult 65+ and forensic mental health) + child and adolescent mental health needs-based grouping | 201,240 | 200,062 |
| **Health of the Nation Scales (HoNOS)** (including working age adults, adults 65+, and those with acquired brain injury, children and adolescent clinician-rated scale, and those with learning disabilities) | 99,959 | 105,260 |
| **Reason for referral code 01** | 42,222 | 44,903 |
| **Reason for referral code 02** | 35,403 | 48,009 |
| **Primary diagnosis** (ICD-10 F2 codes) | 68,132 | 71,858 |
| **First episode psychosis flag*** | 5,288 | 5,709 |
| **Early intervention in psychosis team flag*** (+ reason for referral code 18) | 36,231 | 42,086 |

* Flags are derived from the administrative data and indicate presence of a visit.
